# Supplementary material for: Roles of DNA Methylation in Color Alternation of Eastern Honey Bees (Apis cerana) Induced by the Royal Jelly of Western Honey Bees (Apis mellifera)
Source: Int J Mol Sci. 2024 Mar 16;25(6):3368. doi: 10.3390/ijms25063368 (PMC10969989; doi:10.3390/ijms25063368)
Supplement: Supplementary file 1 [file ijms-25-03368-s001.zip › Table S1.pdf]

Table S1 High quality sequencing data of each sample

Samples	Clean Reads Num	HQ Clean Reads Num(%)	Read Length	Adapter(%)	Low Quality(%)	Poly A(%)	N(%)
CQ-1	44516892	44070934 (99.0%)	150/150	157096 (0.35%)	288862 (0.65%)	0 (0%)	0 (0.0%)
CQ-2	50601692	50400762 (99.6%)	150/150	22592 (0.04%)	178338 (0.35%)	0 (0%)	0 (0.0%)
CQ-3	49541992	49262718 (99.44%)	150/150	27920 (0.06%)	251354 (0.51%)	0 (0%)	0 (0.0%)
NQ-1	59701346	59350214 (99.41%)	150/150	83644 (0.14%)	267338 (0.45%)	0 (0%)	150 (0.0%)
NQ-2	44255848	43986432 (99.39%)	150/150	73990 (0.17%)	195332 (0.44%)	0 (0%)	94 (0.0%)
NQ-3	49021762	48804750 (99.56%)	150/150	31620 (0.06%)	185294 (0.38%)	0 (0%)	98 (0.0%)
