# Supplementary material for: Roles of DNA Methylation in Color Alternation of Eastern Honey Bees (Apis cerana) Induced by the Royal Jelly of Western Honey Bees (Apis mellifera)
Source: Int J Mol Sci. 2024 Mar 16;25(6):3368. doi: 10.3390/ijms25063368 (PMC10969989; doi:10.3390/ijms25063368)
Supplement: Supplementary file 1 [file ijms-25-03368-s001.zip › Table S2.pdf]

Table S2 Data mapped to the honeybee genome

Samples	Total Reads	Mapped Reads	Mapped Ratio%	Genome Size	Sequence Depth
CQ-1	44062202	30941071	70.22	214422117	21.64
CQ-2	50400498	36733405	72.88	214422117	25.70
CQ-3	49262244	34203016	69.43	214422117	23.93
NQ-1	59349720	43176744	72.75	214422117	30.20
NQ-2	43985428	31771053	72.23	214422117	22.23
NQ-3	48804460	35490757	72.72	214422117	24.83
