# Supplementary material for: Roles of DNA Methylation in Color Alternation of Eastern Honey Bees (Apis cerana) Induced by the Royal Jelly of Western Honey Bees (Apis mellifera)
Source: Int J Mol Sci. 2024 Mar 16;25(6):3368. doi: 10.3390/ijms25063368 (PMC10969989; doi:10.3390/ijms25063368)
Supplement: Supplementary file 1 [file ijms-25-03368-s001.zip › Table S3.pdf]

Table S3 Average methylation levels of CG, CHG, and CHH in each sample

Samples	C%	CG %	CHG%	CHH%
CQ-1	0.87	1.40	0.58	0.71
CQ-2	0.87	1.38	0.59	0.70
CQ-3	0.93	1.24	0. 64	0.68
NQ-1	0.76	1.17	0.56	0.61
NQ-2	0.77	1.25	0.56	0.60
NQ-3	0.82	1.39	0.57	0.63
