# Supplementary figures and images for: Roles of DNA Methylation in Color Alternation of Eastern Honey Bees (Apis cerana) Induced by the Royal Jelly of Western Honey Bees (Apis mellifera)
Source: Int J Mol Sci. 2024 Mar 16;25(6):3368. doi: 10.3390/ijms25063368 (PMC10969989; doi:10.3390/ijms25063368)

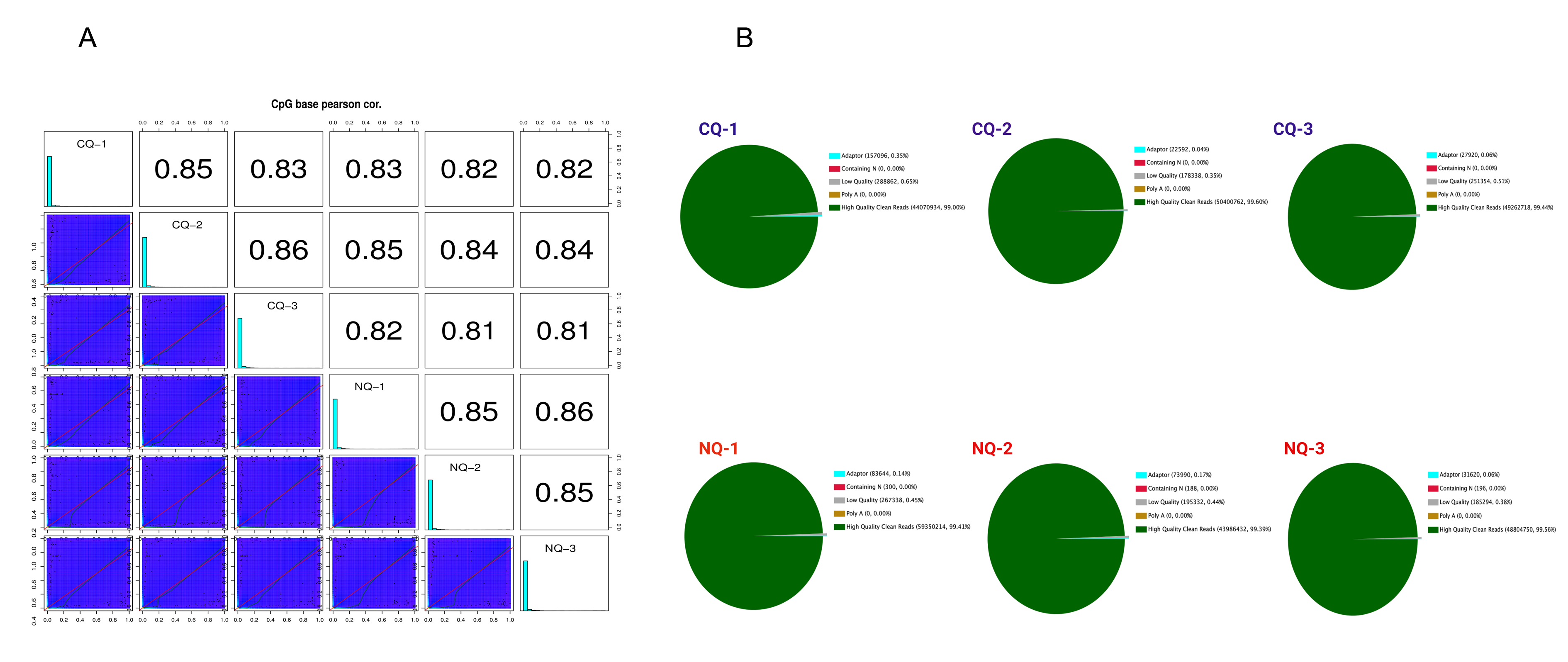

Supplement: Supplementary file 1 [file ijms-25-03368-s001.zip › Figure S1.tif]

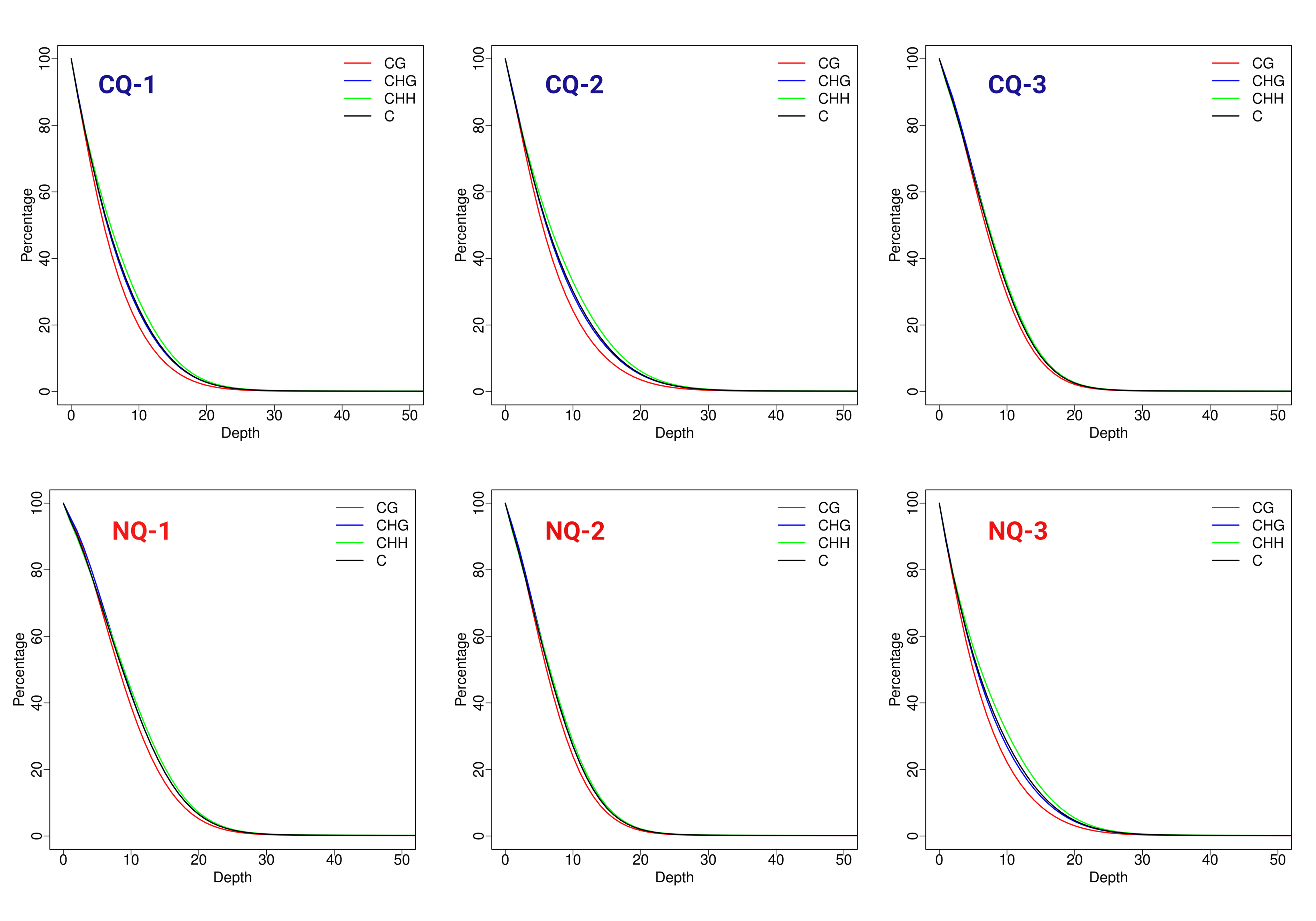

Supplement: Supplementary file 1 [file ijms-25-03368-s001.zip › Figure S2.tif]

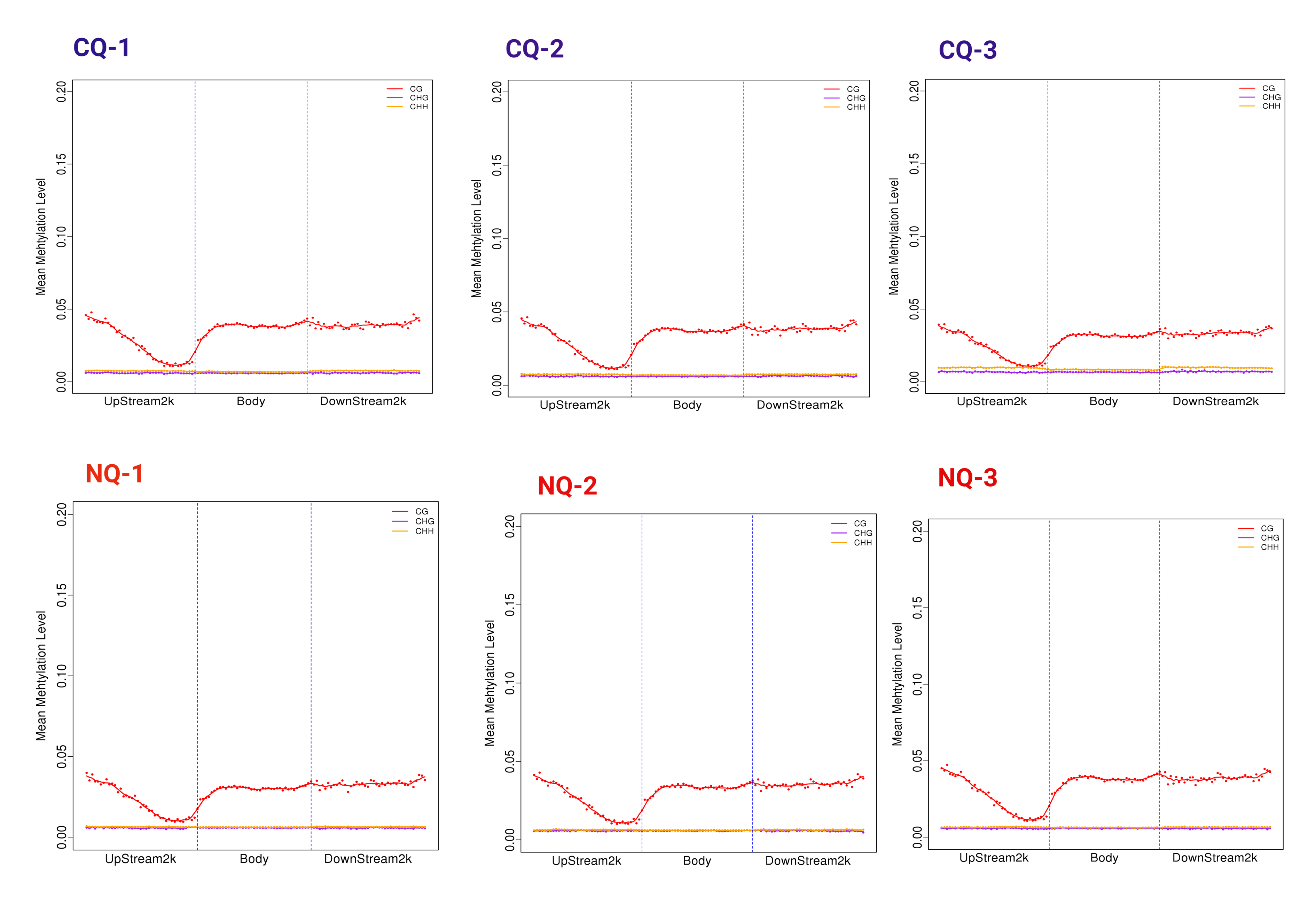

Supplement: Supplementary file 1 [file ijms-25-03368-s001.zip › Figure S3.tif]

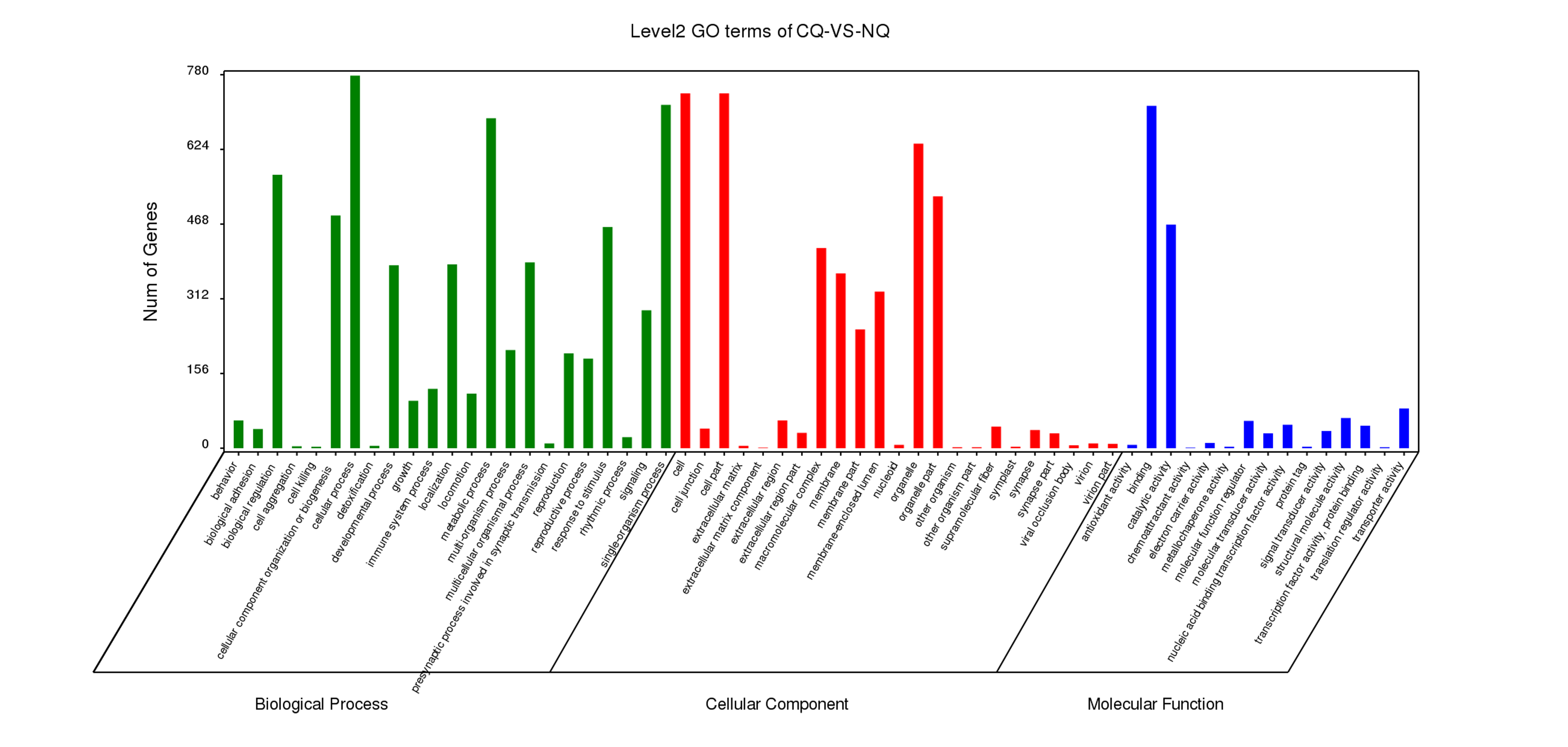

Supplement: Supplementary file 1 [file ijms-25-03368-s001.zip › Figure S4.tif]
